# Introduction to Special Issue of Plant Virus Emergence

**DOI:** 10.3390/v13010055

**Published:** 2021-01-01

**Authors:** Michael Goodin, Jeanmarie Verchot

**Affiliations:** 1 Plant Pathology Department, University of Kentucky, Lexington, KY 40546, USA; 2 Department of Plant Pathology and Microbiology, Texas A&M University, College Station, TX 77802, USA

We are pleased to present in this Special Issue a series of reviews and research studies on the topic of “*Plant Virus Emergence*”. The issue includes a series of articles that elaborate on important plant virus diseases that are among the most recent epidemiological concerns. This Special Issue is predicated on the paradigm that plant virus epidemiology, outbreaks, epidemics, and pandemics parallel zoonotic viruses, and can be consequential to global food security [1]. There is evidence that local, regional, national, and global trade of agricultural products has aided the global dispersal of plant virus diseases. Expanding farmlands into pristine natural areas has created opportunities for viruses in native landscapes to invade crops, while the movement of food and food products disseminates viruses creating epidemics or pandemics [2,3]. As covered in several of the submissions, a number of factors drive plant virus emergence. Included in these are direct anthropomorphic activities, reviewed in “*Homo sapiens: the superspreader of plant viral diseases*” [4]. This theme of distribution of cultivated plants around the world, dispersed from their centers of domestication, implicates humans as being responsible for the novel encounters between plants and their pests. Alternatively, in the article “*Disease Pandemics and Major Epidemics Arising from New Encounters between Indigenous Viruses and Introduced Crops Viruses*” [5], Jones considers the phenomenon of spillover, the movement of viruses from non-cultivated vegetation into introduced crop plants. Such a phenomenon parallels that which occurred in the emergence of Ebola [6], Hendra, and Nipah viruses [7]. Climate change has also created favorable environments for insects that vector plant viruses. Regulatory agencies around the globe are working together to prevent or mitigate the introduction of plant viruses into new areas where they can cause devastation to food security. Moreover, plant virus outbreaks not only directly impact food supply, they also incidentally affect human health. 

Other articles in this Special Issue describe plant viruses that are studied for their associations with “outbreak” phenomena (sudden appearance), reservoir hosts and “spillover” phenomena (transfer from wild to domesticated areas), common genetics, and reliance on “vectors” for viral spread. In “*Potato virus Y emergence and evolution from the Andes of South America to become a major destructive pathogen of potato and other solanaceous crops worldwide*”, Torrance and Talianksly [8] discuss how PVY emerged from the Andes of South America to become a worldwide threat to potato and other solanaceous crops. Due to its broad host range and recombination between strains, PVY and related potyviruses show an episodic emergence of new strains with new virulences. Torrance and Talianksly make the case for research that integrates phylogenetic analyses and high-throughput next-generation sequencing for the detection, identification, and surveillance of new virus strains. Along these lines, Herath et al. present, in “*Family Level Phylogenies Reveal Relationships of Plant Viruses within the Order Bunyavirales*” [9], the importance of phylogenetics in consideration of emergence negative-sense segmented RNA viruses infecting arthropods, protozoans, plants, animals, and humans. Comprehensive phylogenetic analyses of the four “hallmark” genes demonstrated the relatedness of virus species across host eukaryotic species, herein reinforcing the similar emergence phenomena that occur in both plant and animal systems. Furthermore, for these viruses, the link between plants and animals is made by the vectors of the plant-infecting viruses.

Insects are significant drivers of virus emergence, as featured in submissions by Pinheiro-Lima et al., “*Transmission of the Bean-Associated Cytorhabdovirus by the Whitefly Bemisia tabaci MEAM1*” [10], and Schoeny, et al., “*Can Winged Aphid Abundance Be a Predictor of Cucurbit Aphid-Borne Yellows Virus Epidemics in Melon Crop?*” [11]. These studies confirm that we must avoid dogmatic perspectives on plant virus transmission, as the first demonstration of a whitefly-transmitted rhabdovirus. Heretofore unknown virus–vector combinations should be anticipated as drivers of future outbreaks, as demonstrated for *Bemisia tabaci* Middle East-Asia Minor 1 (MEAM1) transmission of bean-associated cytorhabdovirus to common bean, and with a lower efficiency to cowpea and soybean. However, not only is the particular vector important, but the timing of its occurrence in fields relative to crop age can influence emergence. For example, the abundance of *A. gossypii* during the first two weeks after planting is a good predictor of disease caused by CABYV. Early control of the vector is necessary to minimize the potential for CABYV epidemics in melon crops.

Increasingly, scientists depend upon advanced DNA and RNA sequencing technologies to monitor the emergence and spread of new plant virus strains or species, facilitate novel virus discovery, and uncover the etiology of complex diseases. In the article by Weiland et al., “*RNAseq Analysis of Rhizomania-Infected Sugar Beet Provides the First Genome Sequence of Beet Necrotic Yellow Vein Virus from the USA and Identifies a Novel Alphanecrovirus and Putative Satellite Viruses*” [12], advanced sequencing technology was used to discover a complex of viruses underlying rhizomania of sugarbeet. Historically, BNYVV is the acknowledged primary cause of rhizomania however, this work demonstrates the impact on disease severity of co-infecting viruses like beet soil-borne mosaic virus (BSBMV), beet soil-borne virus (BSBV), beet black scorch virus (BBSV), and beet virus Q (BVQ), and a novel Alphanecrovirus.

SARS-CoV-2 is the third coronavirus to emerge in recent years, SARS and MERS being the other two, suggesting that virus surveillance and discovery in reservoir species is important preparatory work for modeling disease outbreaks and planning for vaccines before their emergence in humans. The discovery of emergent virus species and strains requires further molecular characterization. Moodley et al., in “*Emergence and Full Genome Analysis of Tomato Torrado Virus in South Africa*” [13], report new virus genome sequences for ToTV. Wieczorek et al., in “*Development of a New Tomato Torrado Virus-Based Vector Tagged with GFP for Monitoring Virus Movement in Plants*” [14] produce infectious clones needed to study virus infection and molecular interactions with the hosts. Such infectious clone technology is foundational in understanding virus molecular functions, as well as in developing plant viral-based protein expression vectors. It is in this arena that scientists employ plant-based protein expression systems for plant-based vaccine and pharmaceutical production. Developing plant-made antivirals and vaccines is essential to contain and mitigate outbreaks at their earliest outset, thus mitigating recurrences of the tragic events related to the SARS-CoV-2/COVID-19 pandemic. LeBlanc et al. [15] review the state-of-the-art systems for producing “mammalian-compatible” biomolecules in plants, particularly as related to glycosylation.

We hope that you find the collection in this Special Issue informative and of interest. As the public becomes informed of scientific theories on virus disease emergence and spread during the COVID-19 pandemic, it is timely that plant viruses are included in the discussion. We hope that the articles in this Special Issue on “*Plant Virus Emergence*” highlight elaborate efforts in plant virology that support broad models for virus outbreaks, spillovers, genetics, reliance on “vectors” and human trade for spread, as well as the maintenance of viruses in “reservoir” hosts. In closing, we thank all of the authors for their enlightening contributions to this Special Issue.

## Data Availability

Not applicable.
